# *Maaqwi cascadensis*: A large, marine diving bird (Avialae: Ornithurae) from the Upper Cretaceous of British Columbia, Canada

**DOI:** 10.1371/journal.pone.0189473

**Published:** 2017-12-08

**Authors:** Sandy M. S. McLachlan, Gary W. Kaiser, Nicholas R. Longrich

**Affiliations:** 1 School of Earth & Ocean Sciences, University of Victoria, Victoria, British Columbia, Canada; 2 Department of Natural History, Royal BC Museum, Victoria, British Columbia, Canada; 3 Department of Biology and Biochemistry, and Milner Centre for Evolution, University of Bath, Bath, United Kingdom; University of Michigan, UNITED STATES

## Abstract

Mesozoic bird fossils from the Pacific Coast of North America are rare, but small numbers are known from the Late Cretaceous aged sediments of Hornby Island, British Columbia. Most are unassociated fragments that offer little information, but additional preparation of a large coracoid has revealed more details of its structure, as well as three associated wing bones. Phylogenetic analysis suggests that *Maaqwi cascadensis*, gen. et sp. nov. represents a derived crown or near-crown member of Ornithurae, and specifically suggests affinities with Vegaviidae. *M*. *cascadensis* is characterized by large size, and regressions based on dimensions of the coracoid suggest a large bird, with an estimated body mass of approximately 1.5 kilograms. The bones are robust, with thick walls, suggesting that *M*. *cascadensis* was a bird adapted for diving, similar to modern loons and grebes. The wings are short, while the coracoid is unusually short and broad, similar to modern loons. Along with the Ichthyornithes and Hesperornithes, *M*. *cascadensis* and Vegaviidae appear to represent a third clade of bird that evolved to exploit marine habitats in the Late Cretaceous, one specialized for foot-propelled diving and rapid cruising flight over water.

## Introduction

The Upper Cretaceous Nanaimo Group of the Pacific Northwest Wrangellian terrane exhibits considerable faunal diversity. While the outcrops of these marine sediments are extensive, tetrapod material has proven elusive, in stark contrast to abundant and exceptionally well-preserved molluscan fossils (e.g. [1–3]). This is attributable to slope and shelfal depositional environments within the forearc Nanaimo Basin (e.g. [4–6]). Save for a few exceptional examples [7], Nanaimo Group vertebrate material has been largely limited to fragmentary fish material (e.g. [8; 9]). Tetrapod representation has been restricted to isolated bones of dinosaurs [10; 11], a single instance of a pterosaur [12], marine reptiles [7; 9; 13–16], and an assortment of unassociated avian limb bones [17; 18].

The bird diversity remains poorly understood. Only one avian fossil, a large carpometacarpus attributed to the Ichthyornithidae [18], has been assigned to a known group. Strikingly, the Hesperornithes, which are well-represented in the Western Interior Seaway [19–21] and the Canadian High Arctic [22], are not known from these deposits.

Additional preparation of a large avian coracoid, previously attributed to the Ornithurae [18], now reveals further detail of its omal end, as well as adjacent forelimb bones previously hidden in the surrounding matrix. Before preparation, the acrocoracoid appeared to have been lost to erosion but further examination has determined it to be strongly everted ventrally and hidden within the matrix. A humerus, radius, and ulna, found adjacent to the coracoid, lie in positions consistent with deposition as an articulated wing. Unfortunately, erosion has destroyed the shoulder, elbow, and wrist joints.

Bird fossils are extremely rare in the Late Cretaceous of North America, and are almost exclusively recovered as isolated bones (e.g. [18, 22–26]). The discovery of an associated specimen is therefore of interest for understanding the morphology, systematics, and ecology of birds from the end of the Cretaceous.

## Geological setting

In British Columbia, a large part of the southern coast consists of Nanaimo Group rocks from the Wrangellian Terrane, which crop out along eastern Vancouver Island and the Gulf Islands and range southward into the San Juan Islands of Washington State. Traditionally distributed among eleven discrete formations (e.g. [4; 5]), recent work suggests that the Nanaimo Group may consist of a nearly complete, but punctuated, Upper Cretaceous stratigraphic succession ranging from the lower Turonian to the upper Maastrichtian (e.g. [27–29]). Studies of paleomagnetism [30; 31], floral provinciality [32], and detrital zircon provenance [33] have suggested that the Wrangellian Terrane originated at a paleolatitude between 1,600 and 3,500 km south of its present location. Corrections for overlooked compaction suggest that the point of origin is likely to be near the lower value [34; 35] while other studies of biogeography (e.g. [36; 37]) and detrital zircon provenance (e.g. [38; 39]) support the premise that the landmass occupied approximately its current position.

The youngest macrofossiliferous rocks within the Nanaimo Group are those of the Northumberland Formation, which consist predominantly of dark grey mudstones [4; 40; 41; 5]. Exposures of this formation on Hornby Island have produced rich foraminiferan [42–46] and ammonite assemblages [47; 48; 28; 49] which have placed the unit as late Campanian to early Maastrichtian in age. Establishment of the position of magnetochron C32n.2n, which has been done through comprehensive sampling [50–52] and geochemical analysis of carbon isotopes [53], has further refined the age of the section as latest Campanian given the global placement of the Campanian–Maastrichtian boundary [54].

**Fig 1:**
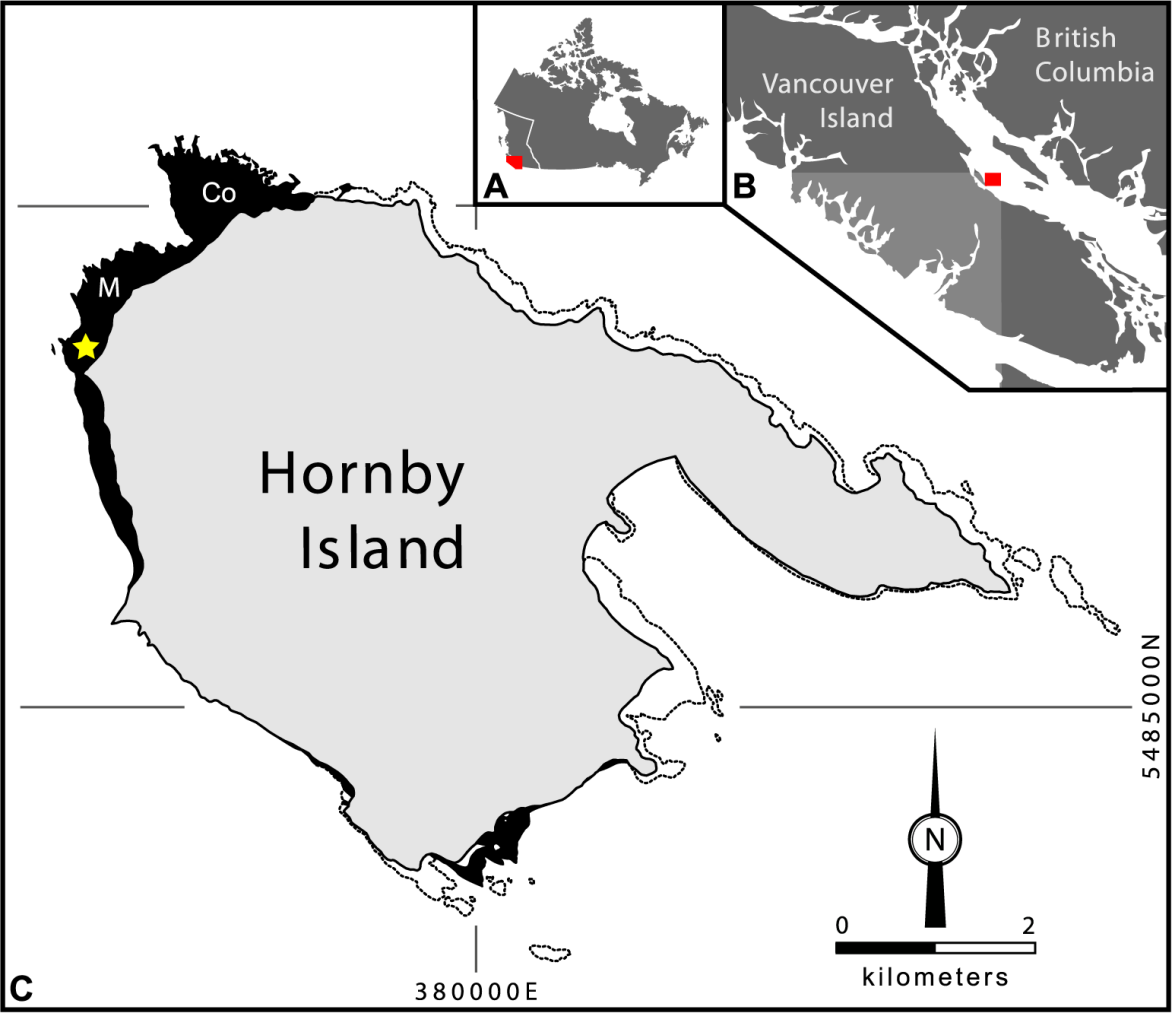
Geographical and geological provenance of *Maaqwi cascadensis* holotype RBCM.EH2008.011.01120. (A) location of the Georgia Basin (red) within British Columbia. (B) location of Hornby Island (red) within the Georgia Basin. (C) intertidal coastal exposures of the Northumberland Formation (black) along Hornby Island adapted from Katnick & Mustard [40; 41]; dotted lines indicate intertidal outcrops of other geological formations; Co = Collishaw Point, M = Manning Point, Star = field locality (49°32'15"N 124°42'50"W). Modified from McLachlan & Haggart [49].

The holotype of *Maaqwi cascadensis*, RBCM.EH2008.011.01120, was recovered along the northwestern shore of Hornby Island (49°32'15"N 124°42'50"W) preserved in a carbonate nodule discovered as float. At this location (Fig 1C), intertidal exposures of the Northumberland Formation have been subjected to coastal erosion and are easily accessible. Contrary to previous reporting [18], field records indicate that the specimen was recovered as float near Phipps Point. This site corresponds to strata 100 m and 200 m down-section of the outcrops at Manning and Collishaw points respectively [49] from which other avian material has been described [17; 18]. With the exception of RBCM.EH2009.021.0001, all of the stratigraphically higher bones were extracted from non-concretionary mudstone matrices within fossiliferous lenses [17; 18].

## Materials and methods

### Systematic paleontology

Avialae Gauthier 1986 [55]
Ornithothoraces Chiappe and Calvo 1994 [56]
Ornithuromorpha Chiappe and Walker 2002 [57]
Ornithurae Haeckel 1866 *sensu* Chiappe [58]
Vegaviidae Agnolín et al. 2017 [59]
*Maaqwi cascadensis* gen. et sp. nov.

### Etymology

The generic name, *Maaqwi*, is derived from “ma’aqwi”, the Coast Salish word meaning “water bird”. The specific name, *cascadensis*, reflects provenance in the Cascadia region of western North America.

### Holotype

**Fig 2:**
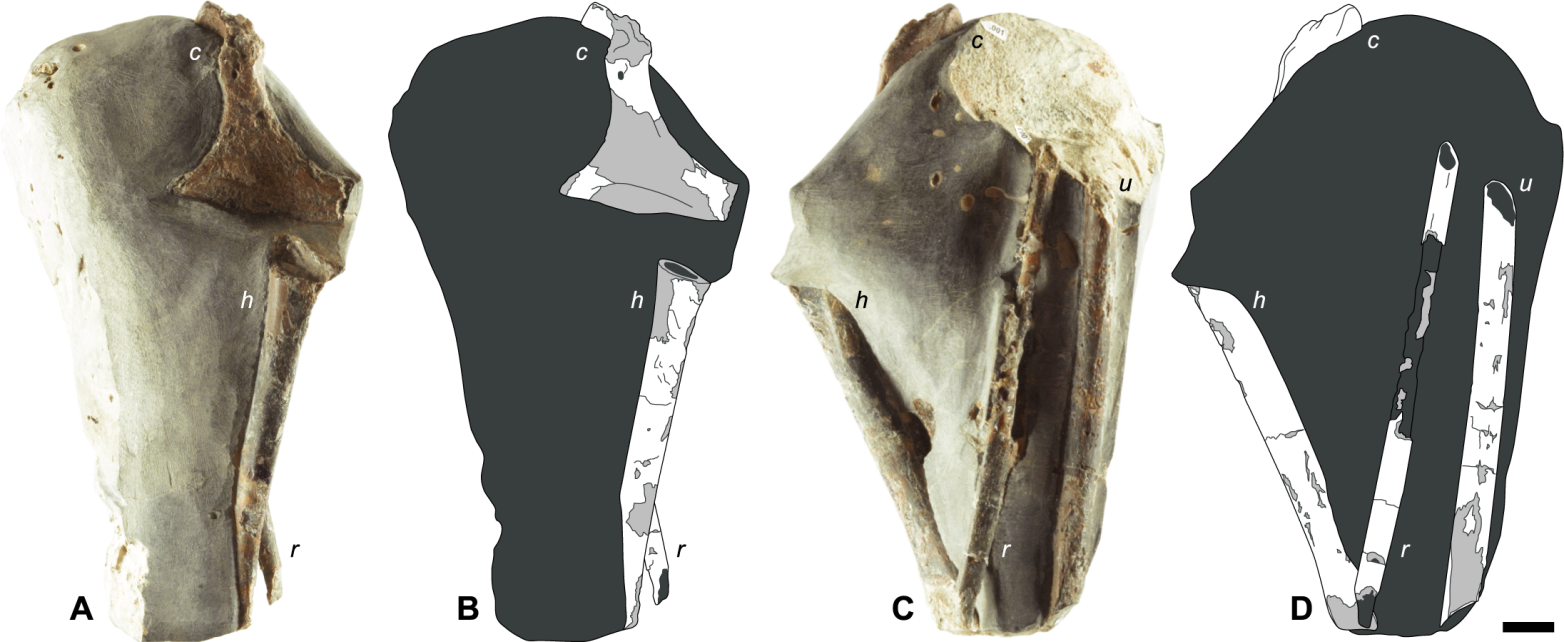
Photographs and schematic illustrations of *Maaqwi cascadensis* holotype RBCM.EH2008.011.01120 depicting wing bone orientation. (A, B) dorsal face of right coracoid and partial humerus. (C, D) matrix rotated 180°; acrocoracoid, partial humerus, ulna and radius. Shading denotes preserved cortical bone (white), exposed trabecular bone (light grey), and matrix (dark grey). *c* = coracoid. *h* = humerus. *u* = ulna. *r* = radius. Scale bar = 1 cm.

RBCM.EH2008.011.01120 consists of a concretionary mudstone nodule containing a right coracoid, as previously described by Dyke et al. [18]. However, at the time of initial description, the specimen had not been prepared and only the dorsal face of the coracoid was visible [18, Fig 2A]. The acrocoracoid appeared to be missing and only the broken ends of the three associated long bones were visible. Subsequent mechanical preparation of the coracoid revealed that its head was everted ventrally and had been buried within the matrix. Further preparation revealed central portions of three wing elements; a humerus, ulna and radius (Fig 2). The specimen is housed within the RBCM.

### Locality

RBCM.EH2008.011.01120 was recovered from a coastal outcrop of the upper Campanian Northumberland Formation exposed on the northwestern shore of Hornby Island, British Columbia.

### Diagnosis

Coracoid compact, polygonal in profile, with the omal portion approximately one third of the medial length. Coracoid shaft a stout, flat bar. Coracoid and humerus robust, highly pachyostotic.

### Description

#### a. Coracoid (RBCM.EH2008.011.01120.001)

The right coracoid is preserved lying in the matrix on its ventral surface. Only a small area near the middle of the shaft shows the original cortical surface of the bone, and much of the articulating surfaces for the humerus and furcula have been damaged by erosion. The total medial length of the coracoid is 42.0 mm, total lateral length is 53.2 mm, and the sternal width is 33.0 mm. The omal length from tip of the acrocoracoid to the base of the procoracoid is 15.7 mm (37.4 percent of the medial length).

Below the procoracoid, the shaft is a flat bar, rather than the cylindrical rod of most other ornithurines. At its midpoint, it is 9.0 mm wide and tapers to 8.1 mm at its narrowest point. It extends for little more than 10 mm before it begins to widen and merges into a broad face of the coracoid as it approaches the sternal articulation. The medial margin of the shaft forms a smooth arc ending in a robust point at the internal distal angle, adjacent to the medial limit of the sternal articulation. The lateral margin of the shaft also sweeps in a smooth arc caudally but ends suddenly at a blunt sterno-coracoidal process. A lateral process appears to be present but it is not a significant structure. In addition to being unusually short and broad, the shaft of the coracoid is also unusual in being highly pachyostotic, with a thick cortex and small medullary cavity.

The acrocoracoid is elevated to the level of the glenoid, as in other Ornithothoraces. It is everted medially, an ornithurine synapomorphy, and strongly wraps around the triosseal canal, as in Neornithes. The medial surface, the *collum acrocoracoidei*, is smooth and unmarked by tendinal scars or grooves. A distinct clavicular articulation is not visible but the distal end is expanded where it presumably would have contacted the furculum. A brachial tuberosity (*tuberculum brachiale*) is discernable.

The lateral surface of the acrocoracoid exhibits a series of grooves and tendinal scars that merge caudally, with a robust ridge that extends caudally along the lateral margin to a point opposite but slightly craniad of the procoracoid. This ridge may mark the dorsolateral margin of the *facies articularis humeralis*. The base of the procoracoid marks the caudal limit of the triosseal canal. It projects medially, to partially extend over the triosseal canal. It appears to have been short and broad, but its distal portions have been damaged by erosion. The *foramen surpacoracodeum* is visible near the base of the remains of the procoracoid, lying medial to nearly invisible remnants of a scapular cotyla. The opening of the foramen lies in a slight depression.

#### Remarks

**Fig 3:**
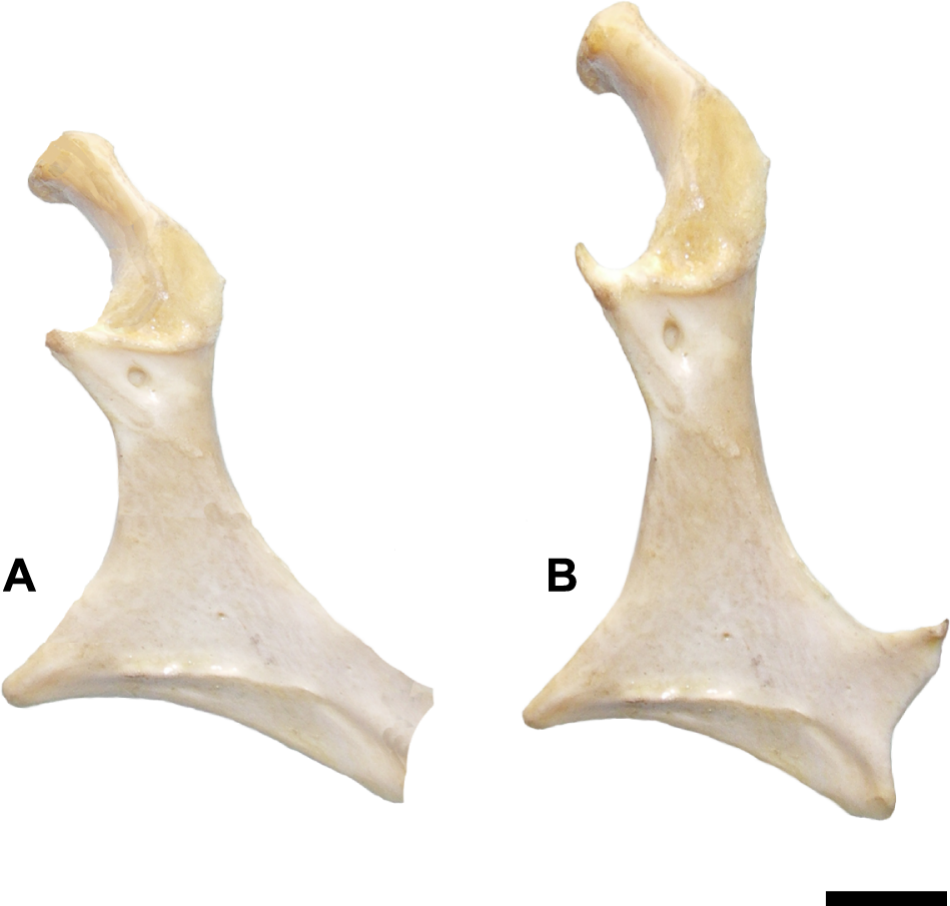
Comparative reconstruction of *Maaqwi cascadensis* coracoid. (A) Digital reconstruction of RBCM.EH2008.011.01120.001; (B) coracoid of Common Loon *Gavia immer*. Scale bar = 1 cm.

Compared to those of other Mesozoic birds, the coracoid of *Maaqwi cascadensis* is very short with respect to either the omal width or the sternal width. The shaft is proportionately shorter and broader than that of basal Ornithurae such as *Ichthyornis* (KUVP 119673) [60] and Palintropiformes [61] or crown-grade ornithurines such as *Cimolopteryx rara* [62]. In comparison to modern birds, it bears a general similarity to the profile of the coracoids in loons (*Gavia* spp.) (Fig 3) or North Pacific albatrosses (*Phoebastria* spp.). However, the sternal articulation is approximately 10 percent longer in proportion to the medial length of the coracoid than in any of the modern families examined.

**Fig 4:**
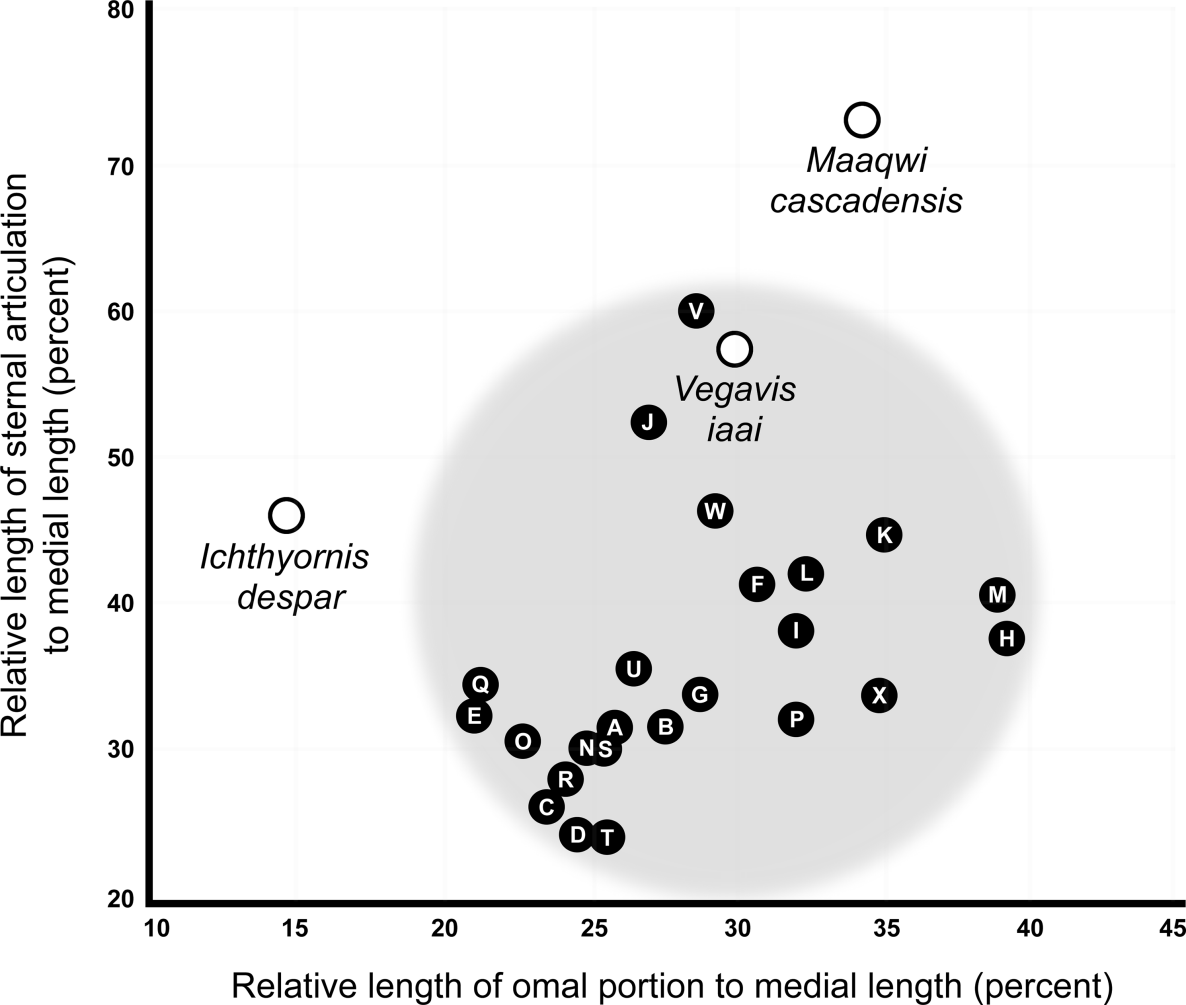
Comparative coracoid morphometrics for some Ornithurine and Neornithine birds. Extant species are indicated by black dots containing a letter: (A) *Aechmophorus occidentalis*; (B) *Alle alle*; (C) *Anhinga anhiga*; (D) *Anhinga melanogaster*; (E) *Ardea herodius*; (F) *Branta bernicola*; (G) *Bubo virginianus*; (H) *Cathartes aura*; (I) *Cepphus grylle*; (J) *Fulmarus glacialis*; (K) *Gavia stellata*; (L) *Grus canadensis*; (M) *Halieetus leucocephalus*; (N) *Larus glaucescens*; (O) *Mergus merganser*; (P) *Pelecanus erythrorhynchos*; (Q) *Phaethon rubricauda*; (R) *Phalacrocorax auritus*; (S) *Phalacrocorax pelagicus*; (T) *Phalacrocorax peniclllatus*; (U) *Podiceps grisigena*; (V) *Puffinus griseus*; (W) *Sula sula*; (X) *Uria lomvia*. Fossil species are named and indicated by open circles.

The proportions of the fossil were compared to measurements from coracoids representing seventeen modern groups of large birds with marine or coastal associations: Alcidae, Anatidae, Anhingidae, Anseridae, Ardeidae, Buteonidae, Cathartidae, Procellariidae, Gaviidae, Gruidae, Laridae, Pelecanidae, Phaethontidae, Phalacrocoracidae, Podicipedidae, Strigidae, and Sulidae; modern materials from which measurements were obtained are housed in the RBCM and the UWBM. Measurements were also included from published figures of *Ichthyornis dispar* [60] and *Vegavis iaai* [63] (Fig 4). These measurements show that the *M*. *cascadensis* occupies an area of morphospace distinct from extant birds or known Late Cretaceous ornithurines. Additionally, none of the coracoids from the modern groups of flying birds showed a comparable degree of pachyostosis.

**Fig 5:**
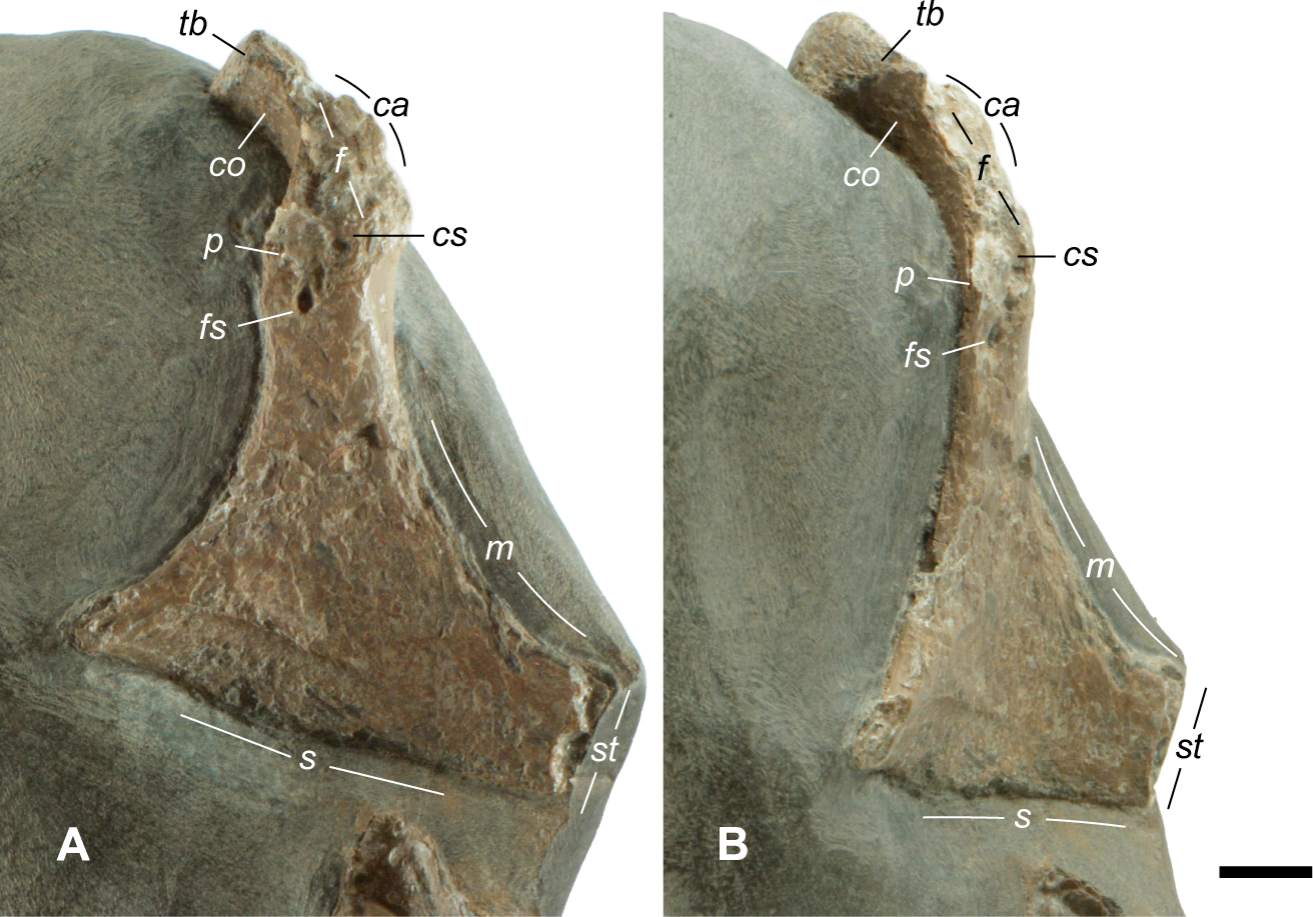
Photographs of *Maaqwi cascadensis* holotype RBCM.EH2008.011.01120 depicting coracoid profile. (A, B) two perspectives of right coracoid RBCM.EH2008.011.01120.001; *ca* = *crista acrocoracoidea* exhibiting surficial erosion, *co* = *collum acrocoracoidei*, *cs* = base of *crista subcapitalis*, *f* = region of *facies articularis humeralis* (*= facies glenoidalis*), *fs* = *foramen supracoracoideum*, *m* = medial margin, *p* = base of *processus procoracoideus*, *s* = sternal articulation *crista articularis*, *st* = sterno-coracoidal process, *tb* = *tuberculum brachiale*. Scale bar = 5 mm.

The omal portion of the coracoid is large—34 percent of the medial length—and is longer than most examples from the modern families examined (Fig 4). It is larger in the Alcidae, Cathartidae, Falconidae, Gaviidae, and North Pacific Albatrosses (*Phoebastria*). Numerous coracoid elements are identifiable following the terminology of Elzanowski et al. [64] employed in their examination of Oligocene procellariifomes (Fig 5). The sternal articulation is entire and uninterrupted, in contrast to some crown Aves, such as albatrosses, where the articulation is divided into distinct medial and lateral portions. A faint ledge along the sternal margin suggests that it fit within a broad notch along the leading edge of the sternum, with a tongue-like lappet of the sternum overlapping onto the coracoid. The ventral margin of the sternum has a sinusoidal ‘M’ shape, with the margin being concave laterally and medially but convex in the center of the articulation.

#### b. Humerus (RBCM.EH2008.011.01120.002)

**Fig 6:**
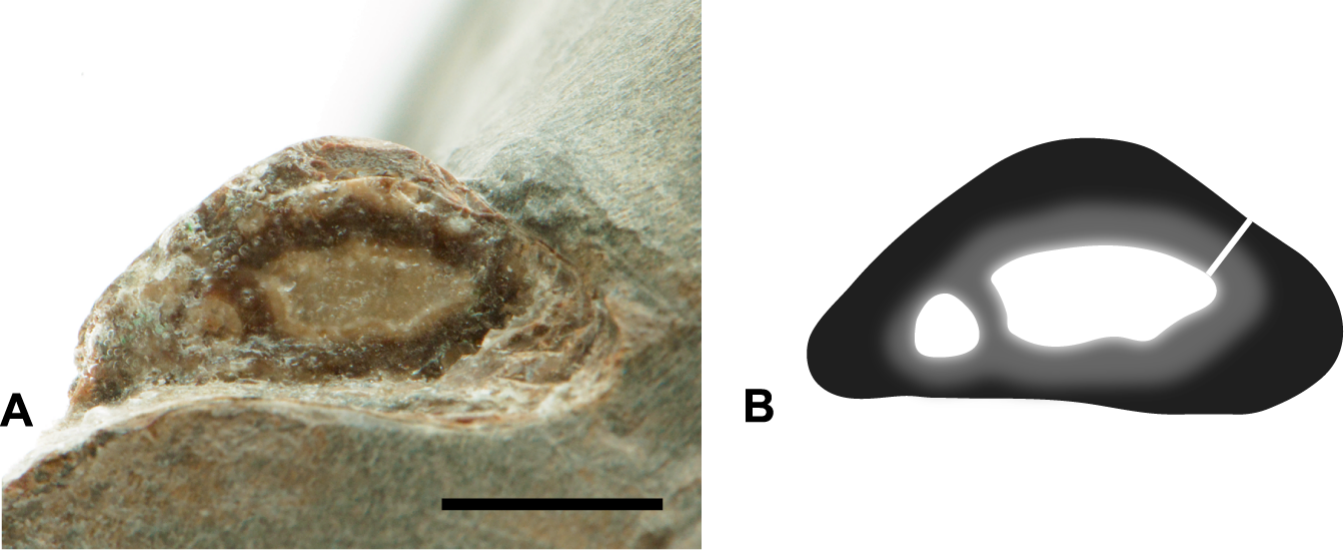
Schematic illustration and photograph of *Maaqwi cascadensis* holotype RBCM.EH2008.011.01120 depicting humerus cross-section. (A) broken proximal end of humerus RBCM.EH2008.011.01120.002 with calcite crystal infill; thickened wall on left is the *crista bicipitalis*. (B) schematic illustration of proximal end of humerus in cross-section; white denotes calcite crystal infill of hollow core; light grey denotes structural discontinuities in the inner layers of bone; white line denotes location of wall thickness measurement. Scale bar = 5 mm.

Part of the right humerus lies in the matrix, near the coracoid. The *caput humeralis*, *crista bicipitalis*, and the *tuberculum dorsalis* are missing. The deltopectoral crest was either not well-developed or the part of the humerus with the deltopectoral crest has been lost. The distal joint has lost all articular structures distal to the epicondyles. The preserved portion of the shaft is 83.5 mm in length and may have been 12–23 percent longer in life. The cross-section of the shaft is slightly oval near its mid-point, measuring 7.7 mm X 6.0 mm. The shaft is relatively straight with no suggestion of the sinusoidal curve seen in many birds. The ends of the bone are strongly flattened. The exposed edges of the bone walls, near the ends of the remaining fossil, vary in thickness (Fig 6) but are massive compared to most flying birds. Maximum proximal thickness is 2.4 mm but a short section of exposed edge near the elbow is only 1.0 mm thick.

#### c. Ulna (RBCM.EH2008.011.01120.003)

The medial surface of the ulna remains in the matrix and, like the humerus, it is missing its proximal and distal ends. The remaining shaft is 85.7 mm long and 6.3 mm wide near its mid-point. Complete, the ulna may have been a little more than 100 mm long. Its cross-section is very close to circular. There is no evidence of remegial papillae. Wall thicknesses at both the proximal and distal ends are about 1.3 mm.

#### d. Radius (RBCM.EH2008.011.01120.004)

The preserved portion of the radius measures 93.0 mm long and 5.4 mm wide. It is more poorly preserved than the adjacent bones, having been damaged by the borings of modern marine endolithic organisms. Its cross-section is circular and wall thickness is between 0.9 and 1.0 mm.

#### Body mass estimates

The dimensions of the coracoid and humerus are strongly correlated with body mass [65] and regression equations derived from modern birds make it possible to make estimates of body mass in fossils of flying birds. From the coracoid, we used minimum shaft width, lateral length, and length of the *facies articularis humeralis*, although the articular facet is badly damaged by erosion (Fig 5) and its measurement may not be reliable. We also measured the circumference of the humerus near its mid-point.

Following the regression equations for flying birds used by Field et al. [65], the measurements of the circumference of the humerus, length of the *facies articularis humeralis*, lateral length of the coracoid, and coracoid shaft width produced body mass estimates of 1.45, 0.98, 1.17, and 2.31 kg respectively [Table 1]. Of these measurements, the length of the humeral articular surface shows the strongest correlation with body mass, followed by humeral circumference, coracoid length, and then coracoid width [65]. However, given the poor preservation of the humeral articular surface, circumference may be more reliable, which would imply a mass of 1.45 kg. Taking the average of all four estimates produces a very similar mass estimate of 1.48 kg. This makes *Maaqwi cascadensis* a relatively large bird, although larger birds, including *Avisaurus*, “Hesperornithiform A”, and “Ornithurine C”, are known from the Late Maastrichtian of North America [62]. Among modern diving birds, *M*. *cascadensis* is comparable to the Red-throated Loon (*Gavia stellata*).

**Table 1:** Body mass estimates for *Maaqwi cascadensis* derived from four parameters. Calculations based on formulae in Field et al. [64].

Parameter	Slope	*y*-intercept	*X*	ln(*x*)	Ln(Mass)	Mass
	(a)	(b)	(mm)			(kg)
**Humerus**						
Mid-shaft circumference	2.51	- 0.81	25.12	3.22	7.28	1.45
**Coracoid**						
Humeral articular surface	2.44	2.00	7.40	2.50	6.88	0.98
Lateral length	3.06	- 5.11	53.42	3.98	7.06	1.17
Shaft width	2.27	3.02	8.02	2.08	7.74	2.31

## Discussion

### Affinities of *maaqwi*

Although the specimen is fragmentary, phylogenetic analysis makes it possible to constrain the position of *Maaqwi* within Avialae, suggesting affinities with derived members of the Ornithurae, and specifically within the recently recognized clade Vegaviidae.

The coracoid is typical of Ornithothoraces in having an elevated acrocoracoid, extending above the level of the scapular articulation in lateral view. The coracoid lacks derived features of Enantiornithes such as a convex lateral articular surface or a scapular articular facet developed as a boss. Instead, the acrocoracoid curves medially to help define a triosseal canal, and has a broad distal end to articulate with the furcula; both are derived features of Ornithurae. Within Ornithurae, the coracoid is similar to *Palintropus*, *Ichthyornis*, and more derived forms in having a ligament scar on the dorsal surface of the acrocoracoid. It is similar to *Ichthyornis* and crown Aves in having a triosseal canal passing ventral to the scapular articular facet. It is more derived than *Ichthyornithes*, but resembles crown Aves in having a humeral articular facet that is displaced anteriorly relative to the scapular articular facet, and an acrocoracoid that is strongly hooked medially to wrap around the triosseal canal.

These features make it possible to identify *Maaqwi* as an ornithurine, as previously suggested by Dyke et al. [18] and to further identify the bird as part of a group of crown or near-crown members of the Ornithurae. These are a grade of birds which, along with *Iaceornis*, *Ceramornis*, *Cimolopteryx*, and a number of unnamed species, show close affinities with the modern avian radiation, but which lack derived features allowing them to be definitively assigned to the crown, or plesiomorphies allowing them to be excluded from the crown [62]. Longrich et al. [62] speculated that these birds might either represent stem Aves just outside of the crown, or else crown birds that lie along the stem of major lineages.

However, in light of recent molecular clock models placing the diversification of crown Palaeognathae, Galloanseres, and Neoaves in the basal Paleogene [66], this greatly constrains the number of places that such Late Cretaceous ‘crown-grade’ birds can conceivably go in the phylogeny. If this model—which implies that only three bird species crossed the K-Pg boundary—is correct, then such ‘crown-grade’ birds must either represent stem Aves just outside of the crown, stem Palaeognathae, stem Galloanseres, or stem Neoaves. If so, then the affinities of *Maaqwi* most likely lie not with extant orders, but with other Late Cretaceous birds, and specifically with Late Cretaceous marine birds.

As discussed, *Maaqwi* appears to lie crownward of Ichthyornithes and Hesperornithes. This leaves a handful of derived ornithurines known from marine habitats, including *Iaceornis*, *Neogaeornis*, *Polarornis*, and *Vegavis*. Aside from representing a Late Cretaceous marine bird of crown-grade, *Iaceornis* [67] shows no particular similarities to *Maaqwi*. *Neogaeornis*, known from a tarsometatarsus [68; 69] and *Polarornis*, known from a partial skeleton [70; 63], have both been interpreted as diving forms. A relationship between *Maaqwi* and these forms seems possible given their shared ecology, although there is no overlap between the fossils. Finally, *Vegavis* has a well-preserved coracoid [71; 63]. It resembles that of *Maaqwi* in being unusually short and broad. Furthermore, a study of the histology has shown that *Vegavis* has pachyostotic long bones, implying that it was a foot-propelled diver [72].

**Fig 7:**
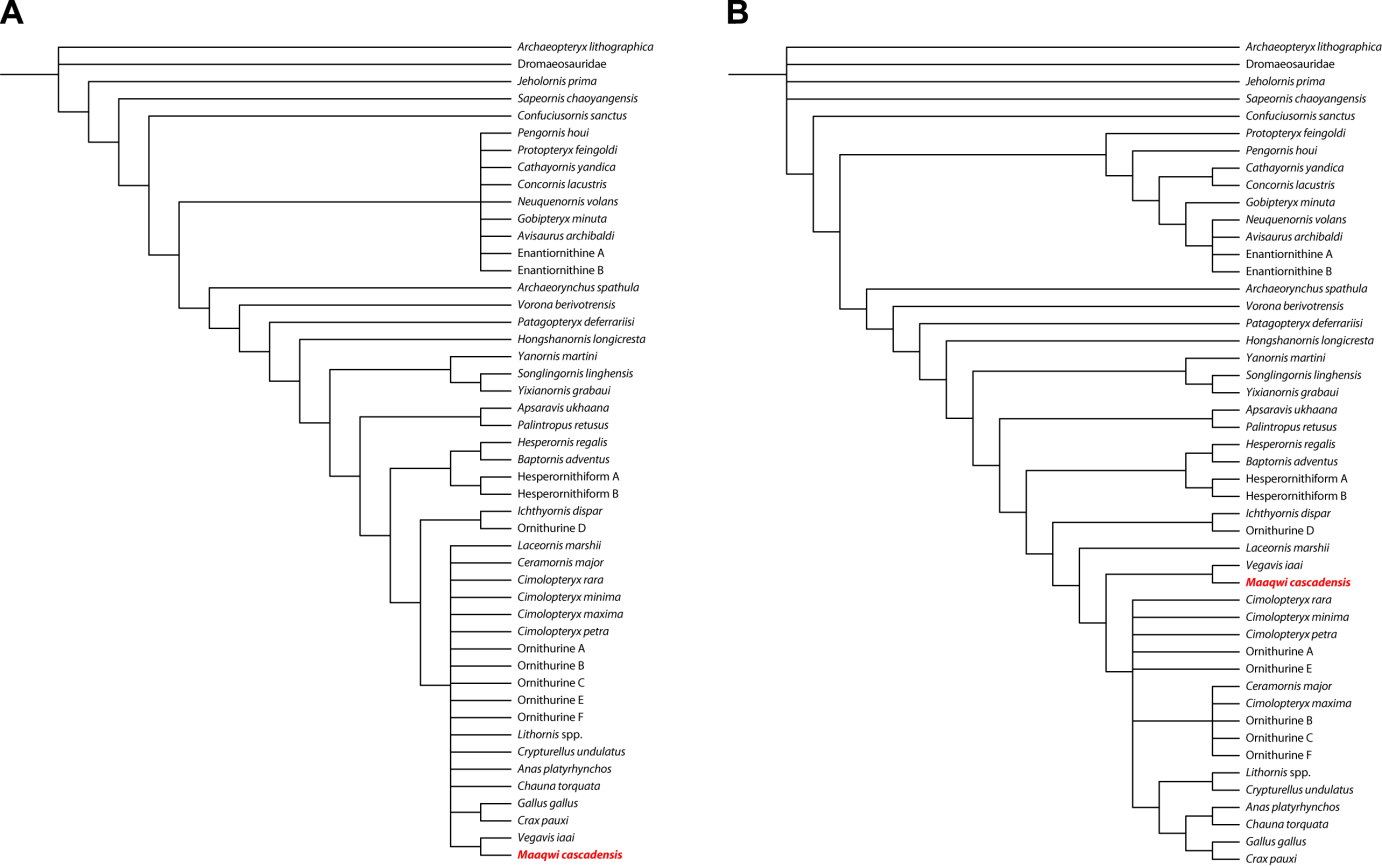
Phylogeny of Mesozoic birds showing the placement of the *Maaqwi cascadensis*. (A) strict consensus of X most parsimonious trees (TL = X, CI = X, RI = X). (B) strict consensus of X most parsimonious trees with minimum ghost range. The Hornby Island bird is consistently recovered as sister to *Vegavis iaai*.

**Fig 8:**
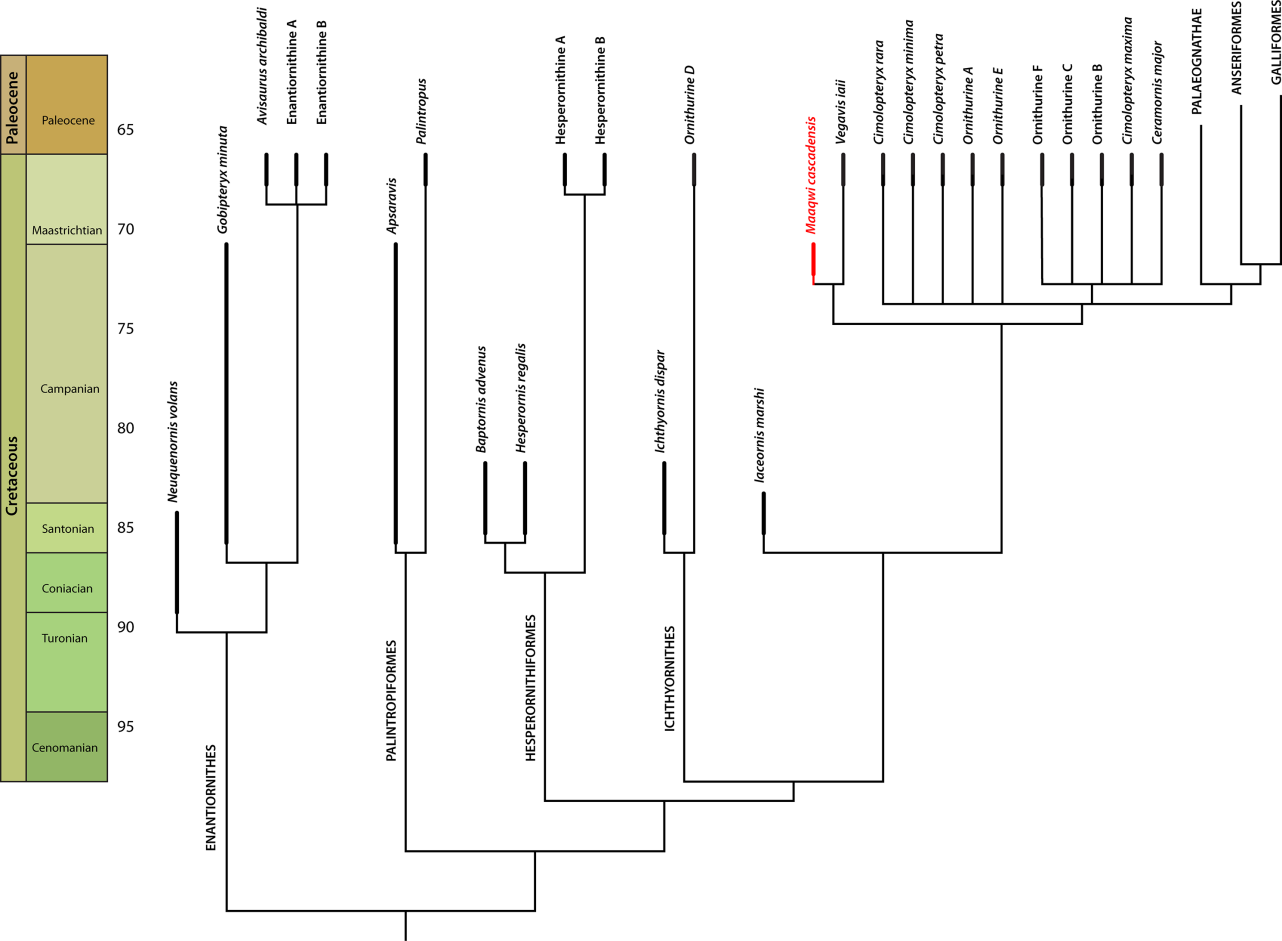
Time calibrated phylogeny showing inferred placement of *Maaqwi cascadensis* as part of a Late Cretaceous radiation of derived Ornithurae.

Addition of *Vegavis* to the coding matrix of Longrich et al. [62] expanded from Zhonghe Zhou et al. [73] and Clarke et al. [74] recovers *Maaqwi* as sister to *Vegavis* (Fig 7) in approach to the terminal Cretaceous (Fig 8). In the current analysis, a single character, the sh ort and broad coracoid, unites them. However, the extreme pachyostosis of the long bones represents another derived character potentially uniting them, and the marine habits represent an ecological synapomorphy, further supporting this result. Although evidence for this hypothesis is currently limited, it appears that *Maaqwi* is most likely related to *Vegavis* as part of a Late Cretaceous radiation of diving birds.

The clade Vegaviidae was recently recognized by Agnolín et al. [59]. According to Agnolín et al., the Vegaviidae includes the Late Cretaceous *Neogaeornis wetzeli*, *Polarornis gregorii*, and *Vegavis iaai*. In addition, the early Paleogene *Australornis lovei* has been recognized as a vegaviid, indicating that the clade crossed the K-Pg boundary. Their analysis differs from the current analysis in recovering Vegaviidae as part of crown Aves and specifically as stem Anseriformes. The analyses differ in their taxon sampling and aim: the Agnolín et al. analysis is specifically designed to assess the ingroup relationships of crown Aves, and therefore might not be appropriate for resolving a taxon that belongs on the stem, while the current analysis is based on a matrix used to assess the relationships of stem birds, and may be inappropriate for resolving the relationships of crown birds. Further study will probably be necessary to better understand the relationships of vegaviids.

### Ecology of *maaqwi*

#### Flight ability

It is unclear whether the *Maaqwi* was capable of flight. The extreme pachyostosis of the long bones would have made the bird relatively heavy for its size, making flight difficult. Furthermore, the wings of *Maaqwi* appear to have been relatively short, implying relatively high wing loading (mass relative to wing area). For example, some albatrosses are comparable to the mass estimated for this fossil (e.g. *Phoebastria nigripes*) [75], but their wing bones are much longer. The preserved portion of the humerus (RBCM.EH2008.011.01120.002) suggests an original length between 90 mm and 95 mm but the humerus of *P*. *nigripes* is more than 270 mm long. The proportions of *Maaqwi* appear to be more similar to loons, which have a high wing loading [76]. The Red-throated Loon, *Gavia stellata*, weighs no more than 2.0 kg [75] and has a humerus about 135 mm long.

The remaining portions of the holotype wing bones suggest that all three limb elements were similar in length, suggesting a brachial index (ratio of humerus to ulna) close to 1.0 [77]. Higher and lower values of the brachial index are indicative of the specialized locomotory capabilities in many extant birds. Soaring, light-bodied frigate birds (*Fregata*) with low wing loading have a brachial index near 0.79 while heavy-bodied loons (e.g. *Gavia immer*) with high wing loading have a brachial index of 1.25 [77]. The brachial index reaches extremes in the Trochilidae (0.69) that use hovering flight and in the Alcidae (1.8) that use wing-propulsion underwater [77]. A high brachial index would suggest that the ulna, which supports much of the airfoil surface, was relatively reduced, consistent with the hypothesis that *Maaqwi* was characterized high wing loading.

The coracoid (RBCM.EH2008.011.01120.001) appears to represent a design that has not been replicated in modern birds, suggesting that it had a distinct flight style. It is unusually short and broad, and aside from a slightly concave area close to the sternal articulation, it is essentially a thick, flat plate. Short, broad coracoids are seen in a number of volant bird lineages, although not to the same degree as in *Maaqwi*. These include albatross and fulmars (Fig 4) and to a lesser degree, loons, auks, and geese. Reduction of the coracoid would have resulted in shortening the pectoralis muscle; since the muscle would contract over a shorter distance, the amplitude of the wingbeat would have been reduced.

Taken together, the relatively short, small wings of the bird, along with the short, broad coracoid, suggest a flight style similar to modern loons, auks, and ducks. These birds are able to fly rapidly and for long distances, in part as a result of their small wings, which reduce drag at high speeds. However, as a result of their small wings, they also have limited maneuverability and often need long taxiing runs to become airborne [76]. As a result, this flight style is most common in birds flying over water, where long takeoff runs are possible and the open environment means that there is little need for maneuvering.

#### Diving adaptation

The coracoid and the humerus of *Maaqwi* exhibit a high degree of pachyostosis, with thickened walls and a reduced medullary cavity. Volant birds have relatively thin-walled bones which act to increase the strength-to-weight ratio, allowing the skeleton to be both light and strong. However, diving birds including auks [78], loons, (e.g. [70]), and especially penguins (e.g. [79]) and plotopterids [80] have thick-walled bones, which act to reduce the buoyancy of the animal. Similar adaptations are seen in aquatic dinosaurs [81] and marine reptiles (e.g. [82]), as well as semiaquatic and aquatic mammals (e.g. [83; 84]).

Although the pachyostosis implies diving habits, it seems unlikely that *Maaqwi* would have been a wing-propelled diver. The bones lack structural characteristics that are typical of modern wing-propelled divers. There is no swelling in the wall of the triosseal gap near the procoracoid [85], the coracoids are relatively short, and the cross-sections of the humerus and the ulna are only slightly oval and not significantly flattened [86].

Flattening of the forelimb skeleton is seen in a range of modern and extinct wing-propelled divers. Flattening of the humerus appears in the limb bones among diving species within the genus *Puffinus* (Procellariiformes, Puffinidae) but not among non-diving species [87]. This adaptation reaches extremes in modern wing-propelled divers such as members of the Spheniscidae, Alcidae, and Pelecanoididae. Flattened wing bones are also seen in Plotopteridae [80], an extinct family of wing-propelled divers from the Oligocene of the North Pacific.

In the absence of any evidence for specialization of the coracoids or the wings, it seems more likely that *Maaqwi* was a foot-propelled diver, similar to loons, grebes, cormorants, and mergansers. Foot-propelled diving is also surmised for the extinct Hesperornithes (e.g. [88–93]) and has been hypothesized for other members of the Vegaviidae including the type genus *Vegavis* [72], as well as *Australornis* [59], *Neogaeornis* [69], and *Polarornis* [70].

## Conclusions

*Maaqwi cascadensis* appears to represent a lineage of Cretaceous marine birds distinct from either Ichthyornithes or Hesperornithiformes. Instead, it appears to be closely allied with—or perhaps part of—crown Aves. The wings are reduced, inconsistent with soaring, and instead suggest a bird specialized for fast cruising flight over water. The thickness of the walls of the bones suggest that it was a diver but the wings are not modified for underwater propulsion. Instead, it was most likely a foot-propelled diver, although it may have made occasional use of its wings for steering underwater. Phylogenetic analysis suggests affinities with *Vegavis iaai*, which has recently been reinterpreted as a foot-propelled diver, taking its place along side other advanced ornithurines specialized for foot-propelled diving within the Vegaviidae including *Australornis lovei*, *Neogaeornis wetzeli*, and *Polarornis gregrorii*. Clearly, additional fossil material is needed to better understand the affinities and ecology of these Late Cretaceous–early Paleogene marine birds.

## Supporting information

S1 File Supplementary information. 1. Coracoid morphometrics. 2. Phylogenetic analysis.
(PDF)

## Abbreviations

KUVP: University of Kansas Museum of Natural History, University of Kansas, Kansas, USA.
RBCM: Royal British Columbia Museum, Victoria, British Columbia, Canada.
UWBM: University of Washington Burke Museum, Seattle, Washington, USA.
