# Supplementary material for: Maaqwi cascadensis: A large, marine diving bird (Avialae: Ornithurae) from the Upper Cretaceous of British Columbia, Canada
Source: PLoS One. 2017 Dec 8;12(12):e0189473. doi: 10.1371/journal.pone.0189473 (PMC5722380; doi:10.1371/journal.pone.0189473)
Supplement: S1 File — 1. Coracoid morphometrics. 2. Phylogenetic analysis. (PDF) [file pone.0189473.s001.pdf]

SUPPLEMENTARY INFORMATION

1. CORACOID MORPHOMETRICS

Institutional Abbreviations

KUVP: University of Kansas Museum of Natural History, University of Kansas, Kansas, USA.

MACN: Museo Argentino de Ciencias Naturales, Buenos Aires, Buenos Aires Province, Argentina.

RBCM: Royal British Columbia Museum, Victoria, British Columbia, Canada.

UWBM: University of Washington Burke Museum, Seattle, Washington, USA.

Comparative Coracoid Morphometrics

Species	Collection Number	*Extremitas omalis coracoidei* (EOC)*	Medial Length	EOC as a percent of Medial Length	Width Sternal Articular Face	Ratio of Sternal Face to Medial Length
**Extant Species**						
American White Pelican	RBCM 20121	42.8	133.3	32.1	43.1	0.32
Anhinga	UWBM 40957	13.3	56.3	23.6	14.8	0.26
Anhinga	UWBM 62930	15.5	62.8	24.7	15.4	0.24
Bald Eagle	RBCM 22394	28.6	73.4	38.9	29.8	0.41
Black Brant	RBCM 10095	16.6	54.1	30.7	22.4	0.41
Black-footed Albatross	RBCM 17354	21.62	54.93	39.4	45.9	0.83

Black-footed Albatross	RBCM 23481	22.51	52.03	43.3	44.5	0.86
Black Guillemot		9.5	29.6	32.1	11.4	0.38
Blue-footed Booby	RBCM 23425	15.3	52.2	29.3	24.2	0.46
Brandt's Cormorant	RBCM 15002	18.7	72.8	25.7	17.8	0.24
Bulwer’s Petrel	RBCM 23392	4.57	15.84	28.9	11.3	0.71
Common Merganser	RBCM 15795	15.6	68.3	22.8	21.0	0.31
Common Murre	RBCM 19183	15.1	43.0	35.0	14.7	0.34
Double-crested Cormorant	RBCM 11279	18.8	77.6	24.3	21.9	0.28
Dovekie		6.0	21.7	27.7	6.9	0.32
Glaucous-winged Gull	RBCM 16055	13.4	53.7	24.9	16.2	0.30
Great Blue Heron	RBCM 23536	16.8	79.5	21.1	26.1	0.33
Great Horned Owl	RBCM 17355	16.0	55.6	28.8	19.0	0.34
Northern Fulmar	RBCM 20080	10.0	37.0	27.0	19.3	0.52
Northern Fulmar	RBCM 19182	11.6	29.94	38.7	17.9	0.60
Pelagic Cormorant	RBCM 23169	14.4	56.5	25.5	17.1	0.30
Red-necked Grebe	RBCM 22377	12.5	47.1	26.5	16.7	0.35
Red-tailed Tropicbird	RBCM 23382	11.6	54.6	21.3	18.9	0.35
Red-throated Loon	RBCM 15301	19.8	56.3	35.1	25.2	0.45
Sooty Shearwater	RBCM 12157	15.54	34.17	45.5	25.4	0.74
Sooty Shearwater	RBCN 12243	12.2	42.7	28.6	25.6	0.60
Sandhill Crane	RBCM 15400	23.2	71.2	32.5	30.1	0.42
Turkey Vulture	RBCM 22387	25.9	65.9	39.3	25.1	0.38
Western Grebe	RBCM 23255	11.4	44.2	25.8	14.1	0.32
**Fossil Species**						
Icthyornis dispar	KUVP 119673	5.5	37.0	14.7	17.0	0.46
Maaqwi cascadensis	RBCM.EH2008. 0011.01120.001	15.7	45.6	34.4	33.2	0.73
Vegavis iaai	MACN-PV 19.748	11.20	37.17	29.71	21.49	0.57

* *Extremitas omalis coracoidei* as defined by Livezey & Zusi [1].

2. PHYLOGENETIC ANALYSIS

Method

Tree projections were generated using Mesquite software version 3.3 (build 854).

Maaqwi cascadensis Character List

Data (bold) was obtained for 26 characters out of a possible 227 following that of Longrich et al. [2] expanded from Zhou et al. [3] and modified from Clarke et al. [4]:

86. Scapula and coracoid: fused (0); unfused (**1**)

87. Scapula and coracoid articulation: pit-shaped scapular cotyla developed on the coracoid and coracoidal tubercle developed on the scapula with 'ball and socket' articulation (**0**); scapular articular surface of coracoid convex (1); scapular articular surface of coracoid flat (2)

88. Coracoid, procoracoid process: absent (0); present (**1**)

89. Coracoid: height approximately equal mediolateral dimension (**0**); height more than twice width, coracoid 'strut-like' (1)

90. Coracoid, lateral margin: straight to slightly concave (**0**); convex (1)

91. Coracoid, dorsal surface (= posterior surface of basal maniraptoran theropods): strongly concave (**0**); flat to convex (1)

92. Coracoid, pneumatized: absent (**0**); present (1)

94. Coracoid, lateral process: absent (0); present (**1**)

96. Coracoid, glenoid facet: dorsal to, or at approximately same level as, acrocoracoid process/'biceps tubercle' (**0**); ventral to acrocoracoid process (1)

97. Coracoid, acrocoracoid: straight (0); hooked medially (**1**)

98. Coracoid, n. supracoracoideus passes through coracoid: present (**0**); absent (1)

99. Coracoid, medial surface, area of the foramen n. supracoracoideus (when developed): strongly depressed (0); flat to convex (**1**)

106. Humerus and ulna, length: humerus longer than ulna (0); ulna and humerus approximately the same length (**1**); ulna significantly longer than humerus (2)

206. Coracoid, glenoid articulation: lateral to scapular orientation (0); anterolaterally oriented (1); anteriorly oriented (**2**)

207. Coracoid, acrocoracoid projecting anteriorly or weakly hooked medially (0); strongly hooked medially (**1**)

208. Coracoid, procoracoid process: medially projecting (**0**) or strongly hooked forward and wrapping around the triosseal canal in dorsal view (1)

209. Coracoid, triosseal canal passing ventromedial to scapular articulation: absent (0) or present (**1**)

210. Coracoid, glenoid projects laterally from body of coracoid as a broad flange: absent (0) present (**1**)

211. Coracoid, shaft straight in lateral view (**0**) or bowed dorsally (1)

212. Coracoid, acrocoracoid medial fossa absent (**0**) or present (1)

213. Coracoid, margin of sternal articulation convex (0) straight or concave (**1**)

215. Coracoid, acrocoracohumeral ligament scar on top of acrocoracoid: absent (**0**) or present (1)

216. Coracoid, medial margin: continuous sheet of bone extending from the sternum to the scapula (0); reduced to a procoracoid process or lost (**1**)

217. Coracoid, simple tab-and-slot articulation with sternum (**0**) or articulation with a tongue-like dorsal process of the sternum (1)

218. Coracoid, medial surface of triosseal canal with a prominent crescentic scar ventrally bounding a fossa: absent (**0**) or present (1)

219. Coracoid, glenoid laterally or dorsolaterally oriented (**0**) or dorsally oriented, lying directly atop the head of the coracoid (1)

Character-Taxon Matrix

Archaeopteryx_lithographica
0000010?000000000??000110??00?0??000?00???000???0010000000000000000?00????????0
000000?00000?0000000000000000?0010000?000??000?00001000000??0000000?00000000010
000000?00000?000000000?00?0?{0 1}1010000000?1{0 1}{0 1}???00?0000000000000000?00
00

Dromaeosauridae
0000000?000000000000000000?00000?000?00??0000???000000000000000000000?100?000000
00(0 1)0000?00000?0000000000000000?0010000?0000000100001000 000??0000000?000000
0001000(0 1)00000(0 1)000(0 1)000000000000000000000000{0 1}00{0 1}{0 1}?0000?00000
00000000000000?0

Jeholornis_prima
???0??????????????????0??????????????10
?10?0?0?0?0?11000?100??0000?00?0???000?1???0???0??1??1001?010000100{0 1}0020?00?0
0??0000????00{0 1}000?0??00?{0 1}00000?0????00??????00??0000??00??00?0??0

Sapeornis_chaoyangensis
1000010000000?????????????????????????????1?0?????0000{0 1}0001001000001 0?0????????
0020000?0000??0?00000000001000?0?01?10?0010000?1???0?00?1{0 1}10?1111001100{0 1}
10?00?{1 2}00?00?0000000???000?010?0??00??0000000?0??11??0???00000??00??00000??0?
0

Confuciusornis_sanctus
11111?(0 1)0010010???????????10??00??011200??01{1 2}0111110010000110010100010(0 1)
020001200001{0 1}000?01000?0??0??0000000000?000211??001000000001010021010 1110?1
00?0001001012010000000000011101011200010001111000011 10111?000-?0000?--1-?000000?
00{4 5}0

Songlingornis_linghensis
{0 1}010010?01????????????????????????????????1??????0?????????????????????31??{1 2}?0
201{0 1}00110110???1?11011???
??0

Patagopteryx_deferrariisi
???????????????????0?0?1??????00??111110000?????????20100000030????????????0????????
?10010????0101?11110{0 1}00???{0 1}?10?0??001000001{0 1}0001??{1 2}00???3?0????????
10?20?0110000?011011?2?0????20?0?000{1 2}11310{0 1}110000000?{0 1}10?00101001?000
0000?0064

Vorona_berivotrensis
??
???1??
?????1010101111200001?20000000??????????????0000000065

Yixianornis_grabaui
101?010001????????????01??1????????11{0 1}?????1?0?????100?2001100301???111031??{1 2
}??20100011011010?1111011111001111 00?0110??001?000???0?011021{0 1}10?3112001100
{0 1}200?00001100000?0001?21?101?2????????1113111{0 1}?1???00??110??0??11?11??0000
0??0{4 5}?

Yanornis_martini
1010010001?01?????????????10????????1????????1?0?????100020011?0301??0???0310?{1 2}???2
0100001011010?1?11?1111000111??0101?0100?10000?0?????1021{0 1}1013111001100{0 1}
200?0??01?000?0?0001??1?????2??0??00?11{2 3}11{0 1}{0 1}?12??00??1?0?????1--1-?00100
0??-{4 5}0

Hongshanornis_longicresta
11110?0?010{0 1}1??????????????10????????????????1{0 1}0?????1?0?{0 1}001100{2 3}01?0?1
11031??{1 2}2??20120001011000?{0 1}010001111011111001010010001 0000?0{0 1}??011001

?1??3112001?00{0 1}200?0??0??0?00?000011?1?101?{1 2}1001000{1 2}1131111112?000?01
?0?00?01??1???01000???{4 5}0
Archaeorynchus_spathula
1?????000{0 1}000?????????????????????????????0?0???01?0?1?00010?{1 2 3}0???????031??
1{1 2}0{1 2}01000010?1{0 1}???0010??111000211??0?01?0??0?10000?????0?10???10??10{1
2}00??0??100?0100??0000??0001?21???1?{0 1}??0??00{0 1}1?1?00?01???00??0?0??0?01??1
???00000??0{4 5}0
Hesperornis_regalis
1110010?1111112001?1?0?1010??1110011100??00?0???10102121011005010011211l?000100
10?0001??0?10?0???000010??0?????????0???0????0?????????????????????????????201?11110
00011111121110212111110021131121222102200 2??-----1??11--1111111164
Baptornis_adventus
{1 2}11???????????????????????????11001??00??1??????????21210110040100112l?{1 2 3}??
012?0??????1010?10?0???000?????0?????????0???0??????????01??{0 1}1{0 1}??????????????
???201?11110000111111211102121111100211211{1 2}1?221?020?21001000?0?1?001111111
164
Pengornis_houi
0000010?00000????????????????????????????????0?????0?{1 2}??0011??{1 2}0??0010?0?????
???012{0 1}001?010??????0??1???012111{0 1}0?011010001{0 1}{0 1}1?????1??11???10{1 2
}{1 2}?0??01??1?11?????0??0??????000??10????{1 2}0?0??0{0 1}?1?1?00??11??11??0?0????
????1???0??00??0?0
Protopteryx_feingoldi
{0 1}00?010?00000????????????10?????????????????0?????0???{0 1}001100{2 3}01?0?10?03
0??1{1 2}0101210011{0 1}10?0?{0 1}010{0 1}?1110001000?0?0000??001??0???????100?01
0?110000??00{0 1}100{0 1}0??0??0000??000??11??????0?????????{0 1}?000?1???11??0?0??
0?????1???-??00?0-?0
Cathayornis_yandica
{0 1}000010?00??{0 1}?????????????????????????????0??????0{0 1}????0111??201?0?10??30
??1??1013100110 11????010?0110000100101100?010001111100{0 1}?1??1???2101210?1?1?0
1?110101201??000000000?11???11{1 2}0?0?0?1{0 1}1?{0 1}?00?????0?????20?00??1??1???0
0000??0{4 5}0
Concornis_lacustris
???0111????????10??30??1?01012100
11011?0?0010001?0???1001?1100?0100?1???10???1{0 1}1??{1 2}????????????????11????{1
2}0?????????00?1???????20?0?00??111?00??1??11??0?0??0??1??1???00000?0040
Neuquenornis_volans
???0110?????????????31?????101{1 2}
1001?01100?001?001?0???2??1???0{0 1}?0???0?????{0 1}???1?11???2???210?1???01???????
???????????????110?1?1?0????????1?{0 1}?0???1?0111??????????1??10???0?00?0?6{3 4}
Gobipteryx_minuta
11111?10010000?001?1?0?10?????00?????0???0??0???1????????????{3 4 5}?????10?????????
??131??1101100?0?1000110?002001?1?0110???0????????????11?121??{0 12}?0??????1?1??1
???????????0?001???0????1?10?00111111000?11?111?0?20?00?0100101100000000 6{4 5}
Avisaurus_archibaldi
???11

01?0???01001???
????????????????????????????????20?0010?001?11????????66
Enantiornithine_A
??11
01??????10??
???????????????????????????????20?00?0??01?11????????66
Enantiornithine_B
??11
01??????1???
???????????????????????????????2??00?????1???????????66
Hesperornithiform_A
??
??
?????????????????01202??1002002??????????????1100011166
Hesperornithiform_B
??
??
????????????????????0???1?0??????????????????11?00?1166
Apsaravis_ukhaana
????1?10??????????????????????????0?1{0 1}?????1?0???????2?20010??4010011???31??1????
?????1001000?0?11001111011111{0 1}0101101000111110000101102111 0?{2 3}11201100?12
??2010111{0 1}0010111111?1110??2?101211211311111?201000?120?010?10-1??0010000?06
{4 5}
Palintropus_retusus
??10
01?0????11?01??
????????????????????????????????20?01?0??11?10????????66
Ichthyornis_dispar
{1 2}110010?11????????0??????10{0 1}1?111011101001020???101?{0 1}1200111?{4 5}1100
21{1 2}?1311022010100001011010?11110111111011110010110110010000101(0 1)10111211
112311201110012?12000111{0 1}00101?11?12111021211111002113112{1 2}2?21000002101
1010111110001000--164
Iaceornis_marshii
??31?02{2 3}11????
1?1011010?111101111?01???????????????????????????????????11131140111012102001110??1
?11?11121110212?121100211???????????????21?10001111100????????64
Ceramornis_major
??10
?1??????11??1???
???????????????????????????????21?10?1??11?00????????66
Cimolopteryx_rara
??10
11110?1111011??
?????????????????????????????????21010001?11100????????66
Cimolopteryx_minima
??10

11?1????11011???
????????????????????????????????21?1000???1?00????????66
Cimolopteryx_maxima
???10
????????11??1???
???????????????????????????????21?10?1?????00????????66
Cimolopteryx_petra
???10
11???????1???
???????????????????????????????21010?0???1?00????????66
Ornithurine_A
???10
11??????110??
???????????????????????????????21010?0?111?00????????66
Ornithurine_B
???10
?1??????110?1??
???????????????????????????????21?10?1??11?00????????66
Ornithurine_C
???10
11??????110?1??
???????????????????????????????21010?1?111?00????????66
Ornithurine_D
???10
11?1????11011???
????????????????????????????????1011010?111?00????????66
Ornithurine_E
???
11010???1?0?1???
????????????????????????????????2??1000???1?00????????66
Ornithurine_F
???10
1???????11011??
???????????????????????????????21010?1??11?00????????66
Lithornis
21111?1121121100?0100011111010110?11{0 1}1101?1111?01?102121012?0{5 6}11?1?1{1 2
}{1 2}13111221001000{0 1}1011011011110111100111110011000 1{1 2}11100001010101101
111113113011110{0 1}12102000?1110110111111211102121011000211311222221100 10(01)21
010?011111000100011067
Crypturellus_undulatus
21111?11211211{0 1}01010001111101011001101111111111001010212101011621011122 1311
112?101000012110110101111111100111110110001211100001110101101111103114011 01112
10201011110110111111211102121021100211311222221{0 1}00101211100011110000100011
067
Anas_platyrhynchos
21111?1021121111111011221102111211111110110001111011212101010621101112131111 31

20110??1011010?1{0 1}111111100001110011000121110000101110111111112311401101012 1021?0111111101111110111011210211002113112{2 3}2221000102210110111111100010001-0 67

Chauna_torquata

11111?10211211{1 2}11110102211011112111111101100011110112121012106211011111311 1140101{0 1}0111011011111111111101021110011000121110000101110110111112311401101 1121021?011111111111111211102121021200211311222222100100200101010111000100011 067

Gallus_gallus

11111?1021121121111011221101111111111111011000110101121210101162100111{1 2}1311 1121101200?12110111111111111111001111011000121110000101110110111111311401100122 1021?01111111111111112111021210211002113112322221001121?110011110000100011167

Crax_pauxi

21111?10211211211110112211011111111111110110001101011212101011621101112131111 21 101200012110111111111111111001111011000121110000101110110111111311401101122102 1 ?011111111111111121110212102110021131123222210011021?10001111100010001106 7

Vegavis

???2????????6????????????????????????10 10010???110?1110?0?11100-10001??-????0???- 2111-2121?2?1???????????????????121110001-11?00???????66

Maaqwi_cascadensis

???10 10010?1?1101??????1??? ?????????????????????????????????21011001?11000????????65

REFERENCES

1. Livezey BC, Zusi RL. Higher-order phylogeny of modern birds (Theropoda, Aves: Neornithes) based on comparative anatomy: I. Methods and characters. Bulletin of the Carnegie Museum of Natural History. 2006; 37: 1–556. doi:10.2992/0145-9058(2006)37[1:PON]2.0.CO;2

2. Longrich NR, Tokaryk TT, Field D. Mass extinction of birds at the Cretaceous-Paleogene (K-Pg) boundary. Proceedings of the National Academy of Sciences. 2011; 108: 15253–15257. doi:10.1073/pnas.1110395108

3. Zhonghe Zhou, Clarke J, Fucheng Zhang. Insight into diversity, body size and morphological evolution from the largest Early Cretaceous enantiornithine bird. Journal of Anatomy. 2008; 212: 565–577. doi:10.1111/j.1469-7580.2008.00880.x

4. Clarke JA, Zhonghe Zhou, Fucheng Zhang. Insight into the evolution of avian flight from a new clade of Early Cretaceous ornithurines from China and the morphology of *Yixianornis grabaui*. Journal of Anatomy. 2006; 208: 287–308. doi:10.1111/j.1469-7580.2006.00534.x
